# Rapid neuralized and vascularized osteogenesis in infected bone defect using biomimetic biomineralized and antibacterial hydrogels

**DOI:** 10.3389/fbioe.2025.1611639

**Published:** 2025-05-21

**Authors:** Yuhao Deng, Song Chen, Maimaitiaili Tuerxun, Xuekang Xiong, Jianfei Tang

**Affiliations:** ^1^ Department of Orthopedic Surgery, Shanghai Sixth People’s Hospital Affiliated to Shanghai Jiao Tong University School of Medicine, Shanghai, China; ^2^ Department of Orthopedic Surgery, Shanghai Sixth People’s Hospital East Affiated to Shanghai University of Medicine and Health Sciences, Shanghai, China; ^3^ Department of Orthopedic Surgery, Jinghong City Hospital Of T.C.M, Dongfeng Management Committee, Jinghong, China

**Keywords:** infected bone defects, bone regeneration, mineralized and antibacterial hydrogel, osteoinductive microenvironment, ostogenesis

## Abstract

Infected bone defects represent one of the most prevalent clinical conditions, affecting millions of patients annually. The local infection and necrosis associated with these defects exacerbate the injury, prolong healing times, and result in significant localized pain, presenting a substantial challenge for clinical repair. In this study, we developed a biomimetic mineralized and antibacterial imCOL1MA hydrogel by employing methacrylated COL1, composite native bone inorganic salts (CNBIS), and Magainin II-PLGA microspheres (mMicrospheres), which was further loaded with bone marrow stem cells (BMSCs) to form osteogenic engineered bone for infected bone defects repair. Briefly, we first optimized the concentration of COL1MA for BMSCs survival, then adjusted proportion of CNBIS to create an appropriate osteoinductive microenvironment, and encapsulated Magainin II in poly (lactic-co-glycolic acid) (PLGA) microsphere for long-term antimicrobial function. Consequently, the promising mineralized and antibacterial imCOL1MA was prepared using 10% COL1MA, 2% CNBIS, and 1% mMicrospheres. The imCOL1MA scaffold served as significant antimicrobial efficacy, excellent biodegradability, good biocompatibility, and osteoinductive microenvironment. As a result, the engineered bone could achieve rapid (only 4 weeks) vascularized and neuralized bone regeneration in a rabbit model of infected bone defects.

## 1 Introduction

Infected bone defects, often caused by trauma, surgical complications, or chronic osteomyelitis, represent one of the most prevalent clinical conditions (Huang et al., 2025; Liu et al., 2025; Yang G. et al., 2025; Zhang et al., 2025b). These defects not only hinder the natural healing processes of osteogenesis, but also lead to functional disability and increased healthcare costs due to recurrent infections, impacting millions of patients annually (Tang et al., 2024; Xu et al., 2025). The local infection and necrosis associated with these infected defects exacerbate the injury, prolong healing times, and result in significant localized pain, presenting a substantial challenge for clinical repair (Tang et al., 2024; Zhang et al., 2025b). At present, the main clinical treatment methods heavily rely on artificial materials implantation, such as metals, ceramics, combining with systemic anti-inflammatory treatment (Ritter et al., 2024; Wang et al., 2024; Shash, 2025). However, due to the non-degradable nature of these artificial materials and the limitation of systemic anti-inflammatory treatment, they frequently fail to address critical issues such as lack of physiological bone regeneration, poor biocompatibility, and multiple complications (Hu et al., 2025; Zhu et al., 2025). Moreover, prolonged the use of antibiotic poses a risk inducing bacterial resistance, while non-degradable implants often lead to temporary structural support and necessary secondary surgeries (Genoud et al., 2025; Sadat Hashemi et al., 2025; Yang Z. et al., 2025). Consequently, these limitations present an urgent need to develop a novel strategy that integrate infection control with functional bone regeneration.

The emergence of tissue engineering technology proposed a promissing approach for infected bone defects repair, combining biodegradable scaffolds, seed cells, biomineralized factors, and antimicrobial microenvironment to achieve physiological bone regeneration (Parthasarathy et al., 2025; Zhang Y. et al., 2025). Yang et al. developed a nanohybrid hydrogel scaffold promoted osteogenesis of infected bone defects (Yang F. et al., 2025). However, the lack of biomineralized microenvironment limited its further application in clinic. Similarly, Xie et al. developed a multifunctional hydrogel to promote infected bone defects repair through the programmed transformation (Xie et al., 2025), but the insufficient osteoinductive microenvironment restrianed its physiological bone regeneration and application. Although Bai et al. constructed a biomineralized hydrogel for bone regeneration (Bai et al., 2024), the lack of antibacterial function hindered its application in infected bone defects. Therefore, developing a multifunctional scaffold that concurrently resists infection, supports cell activity, and mimics native bone biomineralized microenvironment remains a pressing challenge.

Type I collagen (COL1), the primary organic component of bone, plays a critical role in regulating the deposition of inorganic salts, structural and mechanical stability, and the osteogenic differentiation of seed cells (Jiang et al., 2024; Kontakis et al., 2025; Wu et al., 2025; Yanuar et al., 2025). However, pure COL1 is not considered an ideal scaffold due to its unstable state, which caused by the lack of cross-linked network. Similar to the photocrosslinkable hydrogel, the methacrylated COL1 (COL1MA) could address these limitations by offering a stable network, tunable stiffness, rapid gelation, good biocompatibility and biodegradability, and ease of operation, which has been served as promissing basic hydrogel scaffold for bone regeneration (Huang et al., 2025; Zhu et al., 2025). Additionally, most current studies constructed primary hydrogels using methylacrylylated gelatin, silk fibroin, and hyaluronic acid for bone regeneration. Although these hydrogels exhibit photosensitive properties, their fundamental components do not match with the organic constituents of native bone, which might limit their effectiveness in repair bone defects (Spessot et al., 2024; Yu et al., 2025; Zhang et al., 2025a). Biomineralization is one of the most crucial biological processes in bone regeneration, leading it is essential to create an effective biomineralized microenvironment for osteogenesis (Hou et al., 2025; Zheng et al., 2025). It is well known that the mineralized component of native bone tissue is composed of various inorganic salts, including hydroxyapatite, calcium carbonate, calcium citrate, magnesium phosphate, and disodium hydrogen phosphate. This diversity implies that using a single or a limited number of inorganic salts may not provide the optimal regulatory signal for osteogenic induction during mineralization (Bai et al., 2024). Therefore, it is necessary to construct an efficiently biomimetic mineralized microenvironment with total ingredient of composite native bone inorganic salts (CNBIS). Noticeably, bone defects are frequently along with infections, which could seriously hinder the process of bone repair. Therefore, anti-infection therapy is essential in bone defects repair. In comparison to traditional antibiotics and antimicrobial peptides, Magainin II exhibits a broad spectrum of antibacterial activity against various bacteria, which also demonstrated therapeutic effects in fungal and viral infections. Furthermore, its low toxicity and favorable tolerance for patients position Magainin II as an ideal candidate for anti-infective treatment (Koynarev et al., 2024; Zhang L. et al., 2025). By encapsulating Magainin II in poly (lactic-co-glycolic acid) (PLGA) microspheres, sustained release can be achieved, ensuring long-term antimicrobial protection without compromising scaffold bioactivity.

**FIGURE 1:**
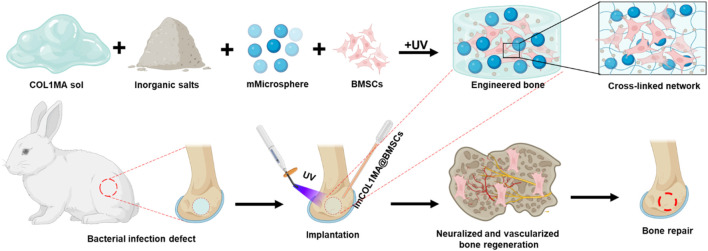
Schematic of rapid neuralized and vascularized osteogenesis in infected bone defect using biomimetic biomineralized and antibacterial engineered bone.

In this study, we developed a biomimetic mineralized and antibacterial dual-functional COL1MA hydrogel (imCOL1MA) by employing methacrylated COL1, CNBIS, and Magainin II-PLGA microspheres (mMicrospheres), which has not been reported so for. The dual-functional COL1MA was further loaded with bone marrow stem cells (BMSCs) to form osteogenic engineered bone for infected bone defects repair (Figure 1). Briefly, we first optimized the concentration of COL1MA for BMSCs survival, then adjusted proportion of CNBIS to create an appropriate osteoinductive microenvironment, and encapsulated Magainin II in poly (lactic-co-glycolic acid) (PLGA) microsphere for long-term antimicrobial function. Consequently, the promising mineralized and antibacterial imCOL1MA was prepared using 10% COL1MA, 2% CNBIS, and 1% mMicrospheres. The imCOL1MA scaffold served as significant antimicrobial efficacy, excellent biodegradability, good biocompatibility, and osteoinductive microenvironment. As a result, the engineered bone could achieve rapid (only 4 weeks) vascularized and neuralized bone regeneration in a rabbit model of infected bone defects. Therefore, this strategy proposed a significant advancement for addressing the multifaceted challenges of infection-related bone defects, offering a clinically translatable method for rapid vascularized and neuralized bone repair. Consequently, a biomimetic mineralized and antibacterial dual-functional imCOL1MA hydrogel scaffold was successfully developed, which showed stronger promising for clinical translation compared to traditional biomaterials that focus only on biomineralization or antibacterial properties (Bai et al., 2024; Tang et al., 2024).

## 2 Results and discussions

### 2.1 The concentrated selection of COL1MA

**FIGURE 2:**
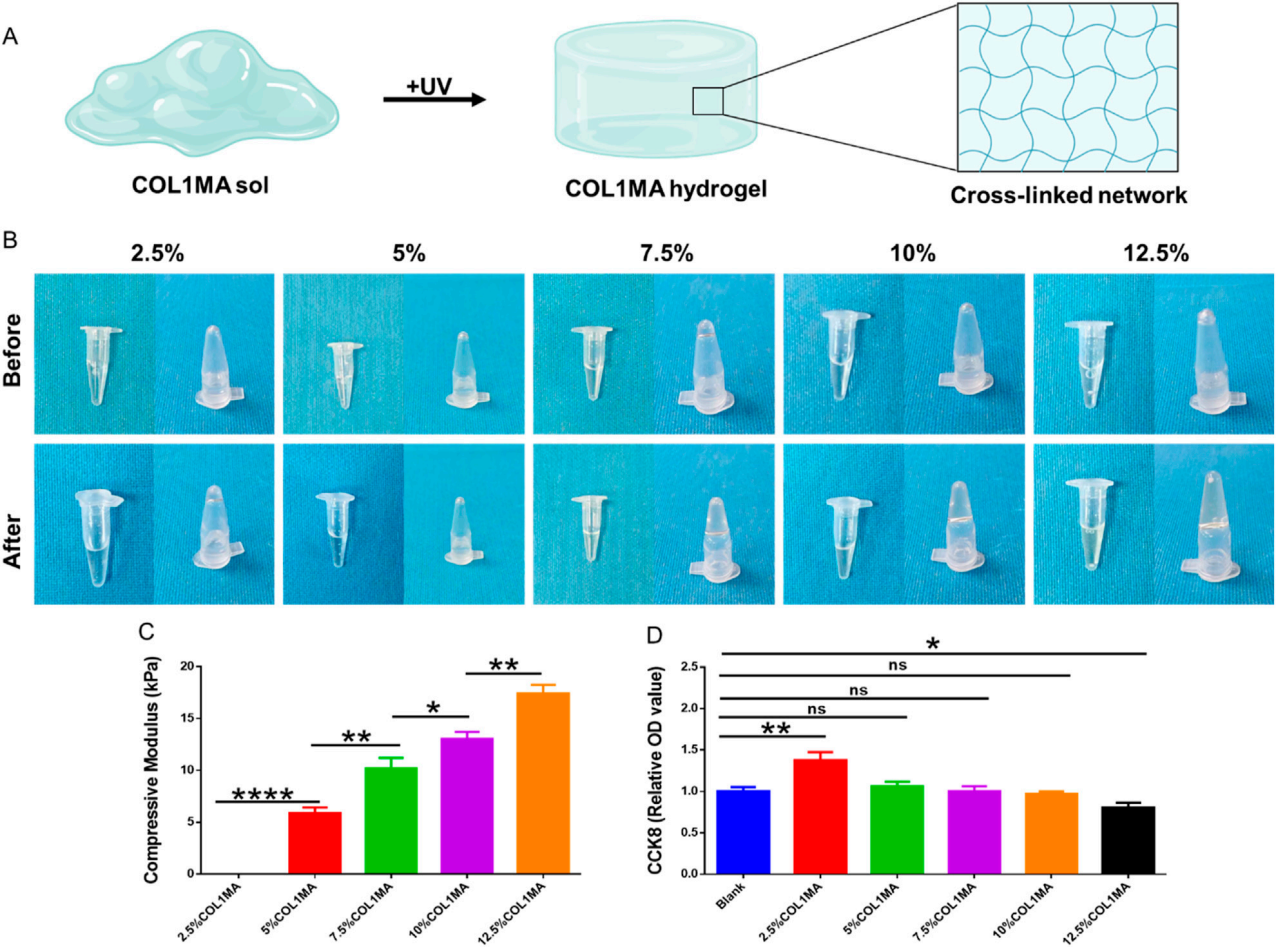
The concentration selection of COL1MA. **(A)** Schematic diagram of COL1MA preparation. **(B)** Gross views before and after photocrosslinking in 2.5%, 5%, 7.5%, 10%, and 12.5%COL1MA. **(C)** The compressive modulus of 2.5%, 5%, 7.5%, 10%, and 12.5%COL1MA. **(D)** Cell proliferation on 2.5%, 5%, 7.5%, 10%, and 12.5%COL1MA was assessed using CCK8.

The biomechanics of scaffolds have been regarded as one of critical factors in influencing the survival, proliferation, and differentiation of seed cells with the advancement of regenerative medicine and tissue engineering (Baratchi et al., 2024; Zauchner et al., 2024; Peng-Fei et al., 2025). The mechanical properties of the COL1MA hydrogel scaffold are closely linked to its cross-linked network and concentration. Thus, it is essential to select a COL1MA concentration that is optimal for seed cell growth to ensure the successful repair of bone defects. In this study, we assessed the rheological properties of the COL1MA hydrogel at concentrations of 2.5%, 5%, 7.5%, 10%, and 12.5% (Figures 2A,B). The results indicated that the compressive modulus increased with rising of COL1MA concentration (Figure 2C). However, COL1MA concentrations exceeding 10% adversely affected cell proliferation (Figure 2D). Therefore, considering the practical applicability and the aforementioned findings, we selected 10% COL1MA as the foundational hydrogel scaffold.

### 2.2 The proportion adjusting of CNBIS

**FIGURE 3:**
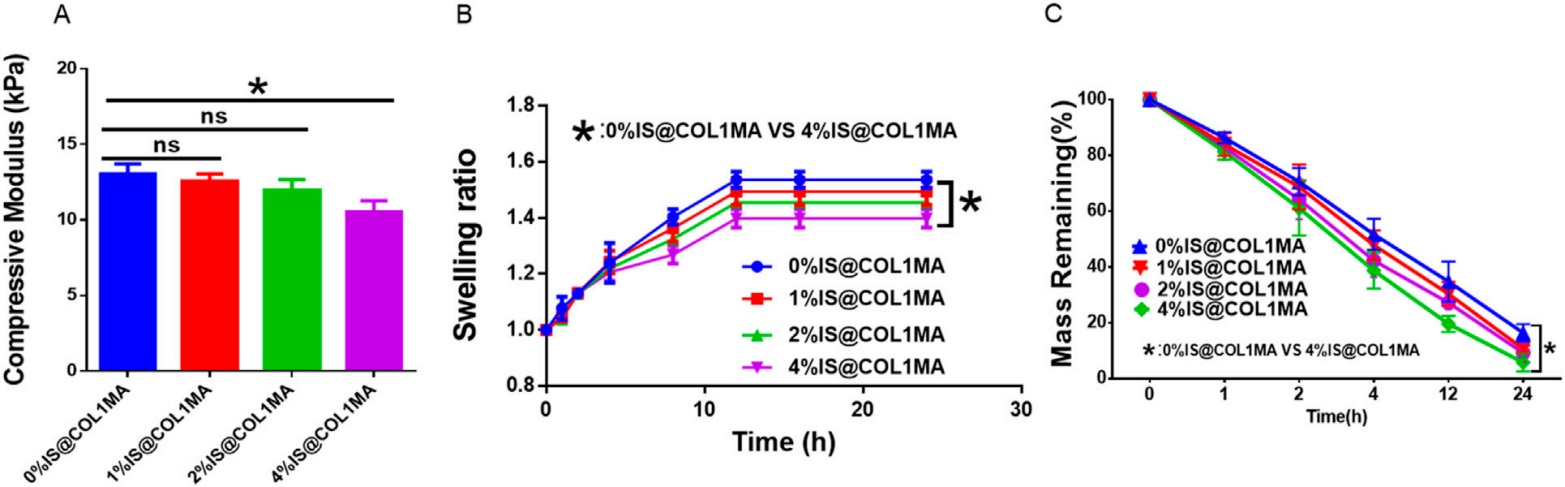
Proportional selection of CNBIS. **(A–C)** The compressive modulus **(A)**, Swelling ratio **(B)**, and degradation rate of 0%IS@COL1MA, 1%IS@COL1MA, 2%IS@COL1MA, and 4%IS@COL1MA.

Biomineralization is one of the most complex and crucial biological processes in bone regeneration, leading it is essential to create an effective biomineralized microenvironment for osteogenesis (Bai et al., 2024). Obviously, a single type or a limited number of inorganic salts cannot fulfill the complex mineralized microenvironment necessary for efficient bone regeneration. Currently, inspired by the composition and proportion of native bone inorganic salts, we prepared composite native bone inorganic salts (CNBIS) by mixing hydroxyapatite, calcium carbonate, calcium citrate, magnesium phosphate, and disodium hydrogen phosphate in the proportions of 84%, 10%, 2%, 2%, and 2%, respectively, to construct a biomimetic and effective native mineralized microenvironment. However, the amount of CNBIS added to COL1MA would affect its mechanical strength, swelling, and degradation properties. To determine the optimal amount of CNBIS addition, we established groups with 0%, 1%, 2%, and 4% CNBIS, and measured their compressive modulus (Figure 3A), swelling (Figure 3B), and degradation (Figure 3C). The results indicated that the addition of 4% CNBIS significantly influenced the characteristics of the COL1MA hydrogel. Consequently, the iCOL1MA hydrogel with a biomimetic mineralization microenvironment was constructed by incorporating 2% inorganic salts into the COL1MA hydrogel.

### 2.3 The antibacterial properties and characterizations of imCOL1MA

**FIGURE 4:**
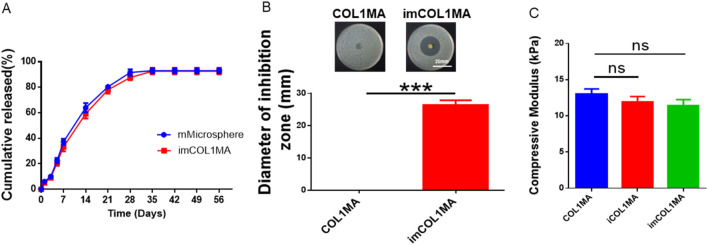
Evaluation of antibacterial properties. **(A)** Cumulative release of Magainin II in mMicrosphere and imCOL1. **(B)** Gross views and quantitative analysis of the inhibition zone. **(C)** The compressive modulus of iCOL1MA and imMCOL1MA groups reveled that the addition of Magainin II and microsphere would not influence the characterization of the hydrogel.

**FIGURE 5:**
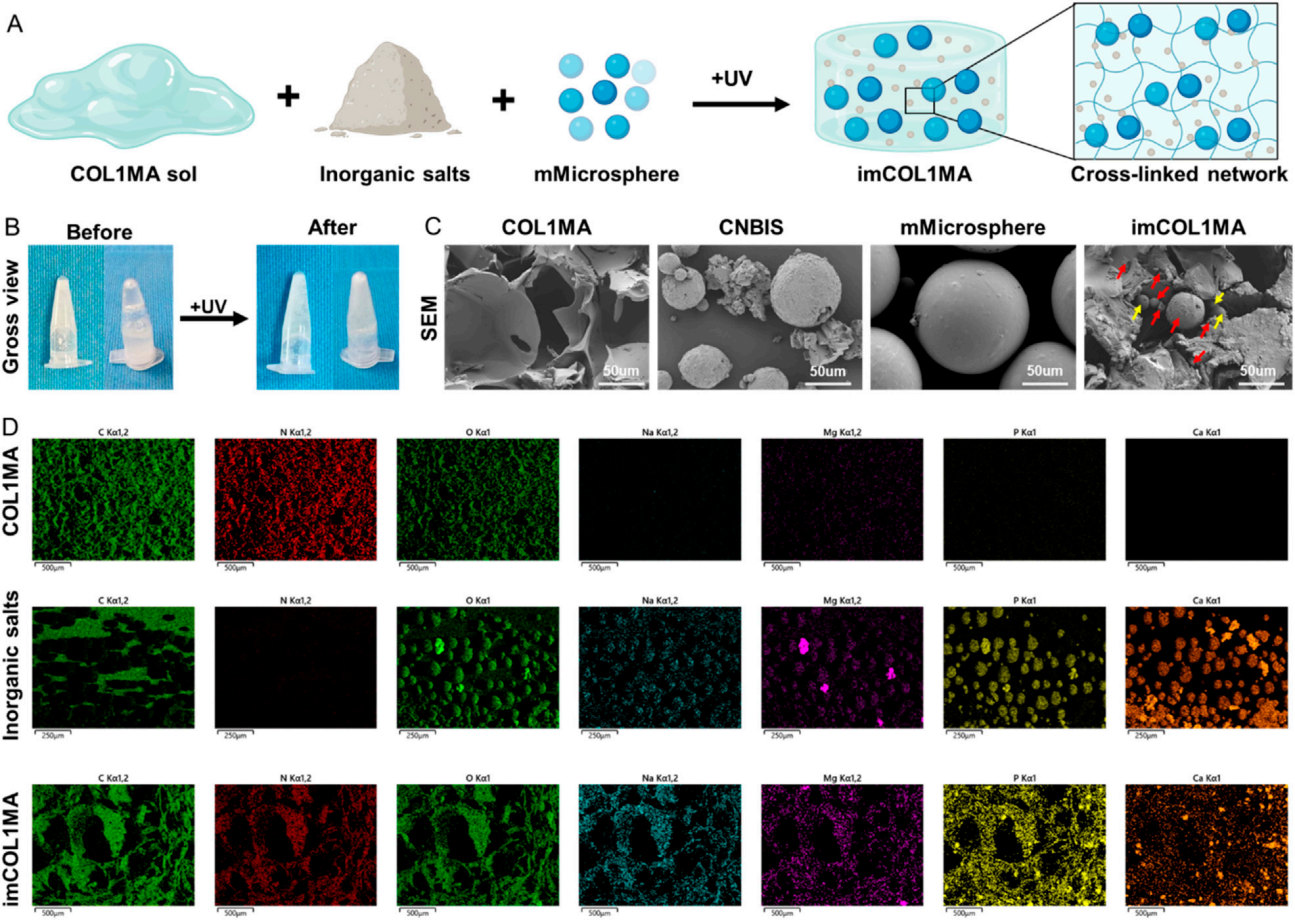
Characterization analysis of the imCOL1MA. **(A)** Schematic diagram of imCOL1MA construction. **(B)** Gross views before and after photocrosslinking imCOL1MA. **(C)** SEM of COL1MA, inorganic salts, mMicrosphere, and imCOL1MA. The red arrows represent CNBIS and the yellow arrow represents the mMicrosphere. **(D)** Elemental analysis of carbon, nitrogen, sodium, magnesium, phosphorus and calcium in COL1MA, inorganic salts, and imCOL1MA groups.

Infection-related bone defects proposed a great clinical challenge with high morbidity, affecting millions families globally annually (Huang et al., 2025; Liu et al., 2025). These infected bone defects not only hinder the natural healing processes of osteogenesis, but also lead to prolonged pain, functional disability, and increased healthcare costs due to recurrent infections. To address the aforementioned chanlleges, we identified Magainin II, a broad-spectrum antimicrobial peptide, as the antimicrobial agents due to its advantages over conventional antibiotics, including rapid bactericidal activity and a low risk of resistance. To achieve long-term antimicrobial function, we encapsulated Magainin II in poly (lactic-co-glycolic acid) (PLGA) microsphere (mMicrosphere). The results revealed that the mMicrosphere could release Magainin II more than 28 days with over 80% release (Figure 4A), indicating that the antibacterial action could accompany the whole process of bone regeneration. Moreover, methicillin-resistant *Staphylococcus aureus* (MRSA) infection is the main reason of clinically refractory infection. In order to explore the effect of the hydrogel against MRSA, we set the COL1MA as the control, and the mMicrosphere-loaded iCOL1MA (imCOL1MA) as the experimental group to verify the antibacterial function (Figure 5A). The results showed that imCOL1MA exhibited a significant antibacterial effect (Figure 4B). Additionally, the addtion of mMicrosphere not significantly influence its mechanical strength (Figure 4C). Furthermore, the rheological properties of imCOL1MA demonstrated its reliable crosslinking and a mild influence from the added CNBIS and mMicrosphere (Figure 5B). The Scanning electron microscopy (SEM) images showed the structures of COL1MA, CNBIS, mMicrosphere, and imCOL1MA (Figure 5C). Elemental analysis of carbon (C), nitrogen (N), oxygen (O), sodium (Na), magnesium (Mg), phosphorus (P), and calcium (Ca) in COL1MA, CNBIS, and imCOL1MA groups, indicating the successful introduction of CNBIS in the imCOL1MA scaffold (Figure 5D).

### 2.4 Biocompatibility and biological function of imCOL1MA

**FIGURE 6:**
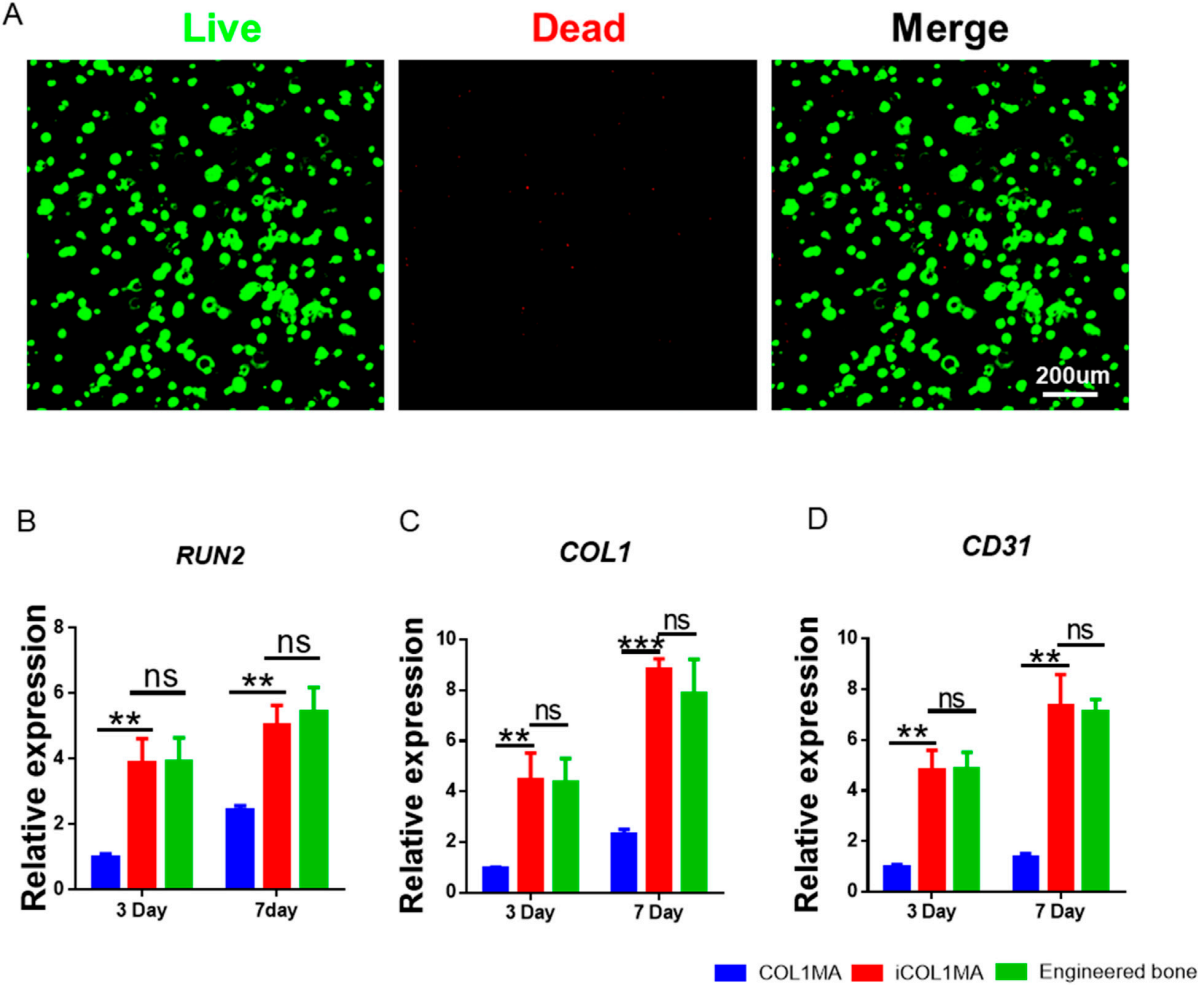
Biocompatibility of imCOL1MA. **(A)** Live (green) and dead (red) staining of BMSCs loaded imCOL1MA. **(B–D)** The osteogenic-related genes expression level of *RUNX2* **(B)**, *COL1* **(C)**, and *CD31* **(D)**.

Good biocompatibility is a fundamental requirement for tissue regeneration scaffolds, as it determines whether the loaded cells can survive and perform their functions effectively. Additionally, good biocompatibility also ensures that the scaffold dose not induce local toxicity after transplantation *in vivo*. In order to investigate the biocompatibility of imCOL1MA, we loaded BMSCs into imCOL1MA scaffolds and cultured for 7 days to evaluate live and dead cell (Figure 6A). The results showed that the cells survived well with only a few dead cells, indicating that the scaffolds exhibit good biocompatibility. In addition, we further evaluated the biological functions of the scaffolds by RT-qPCR, whose results showed that the osteogenesis-related genes in iCOL1MA group were significantly higher than that of COL1MA group (Figures 6B–D), indicating that the addition of CNBIS significantly enhanced the expression of osteogenic genes. Moreover, there was no difference in osteogenic gene expression between iCOL1MA and imCOL1MA groups (Figures 6B–D), indicating that the microenvironment for osteogenesis was not influenced by the addition of mMicrosphere.

### 2.5 Repair of infected bone defect using biomimetic biomineralized and antibacterial engineered bone within 4 weeks

**FIGURE 7:**
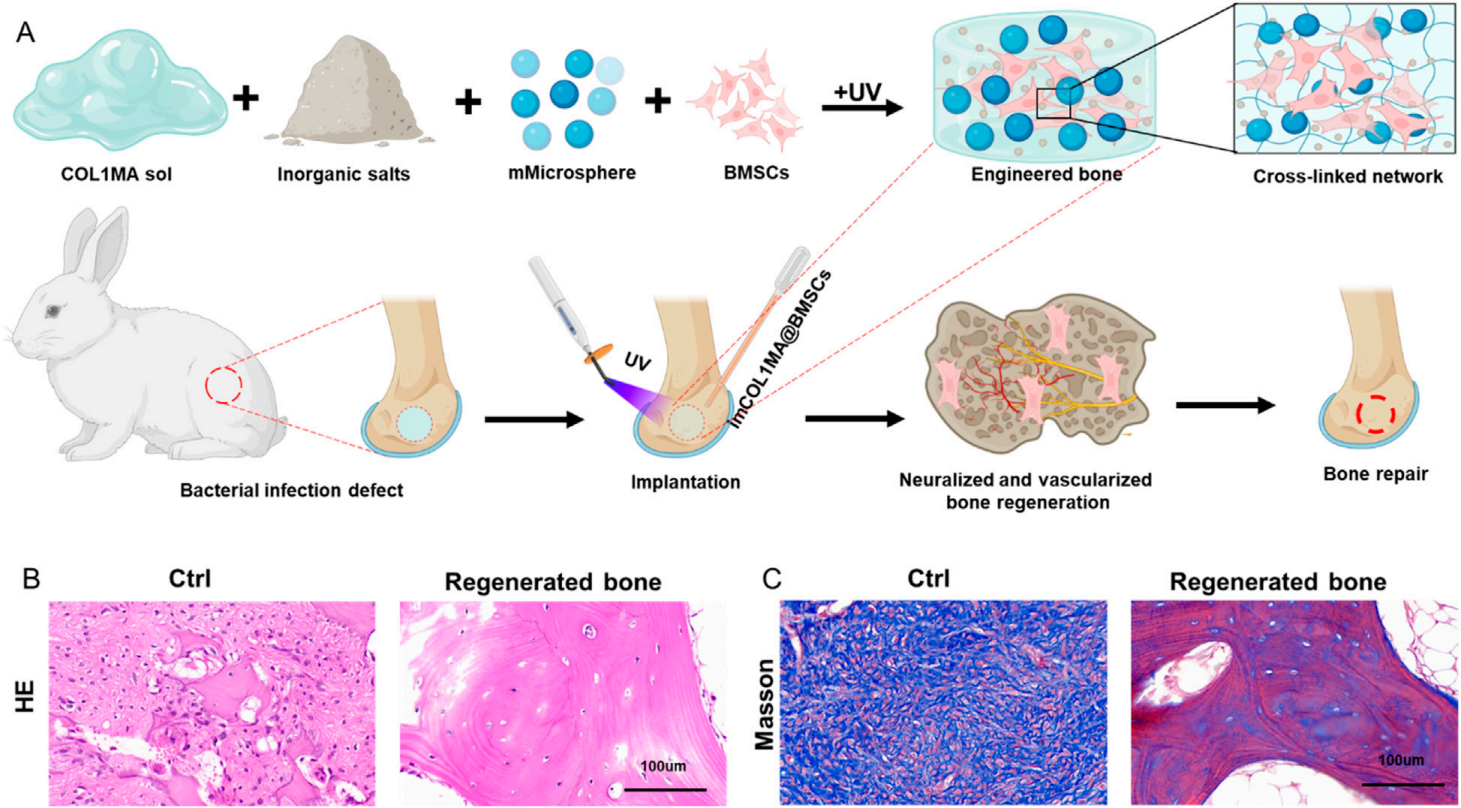
Repair of infected bone defect using biomimetic biomineralized and antibacterial engineered bone. **(A)** Schematic of rapid osteogenesis with neurogenesis and angiogenesis in infected bone defect using biomimetic biomineralized and antibacterial engineered bone. **(B–C)** The H&E **(B)** and masson **(C)** staining in Ctrl and engineered bone groups.

**FIGURE 8:**
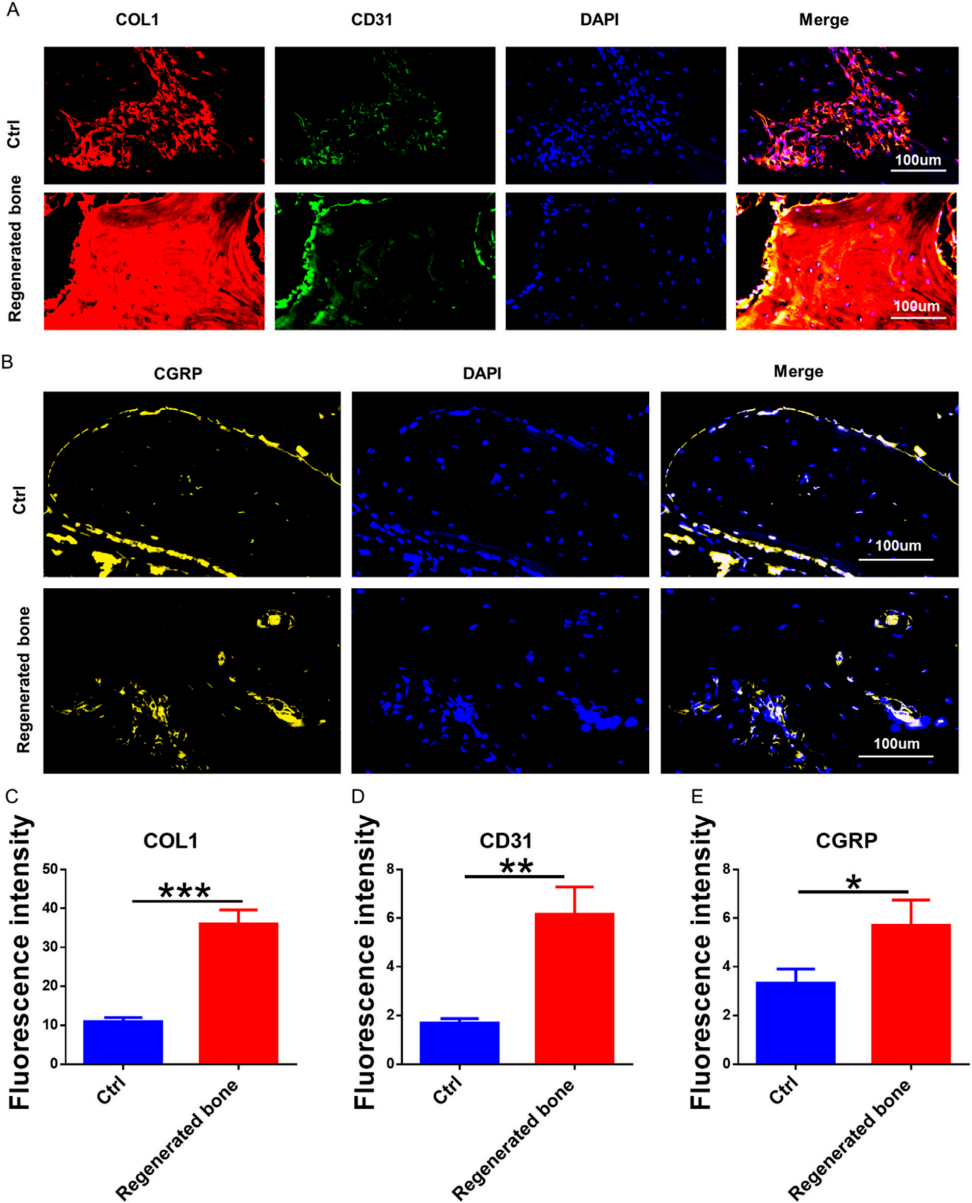
The neuralized and vascularized bone regeneration. **(A)** The bone specific marker (COL1) and blood vessels specific protein (CD31) staining. **(B)** The immunofluorescence staining of neuralized specific marker. **(C–E)** The fluorescence intensity of COL1 **(C)**, CD31 **(D)**, and CGRP **(E)**.

**FIGURE 9:**
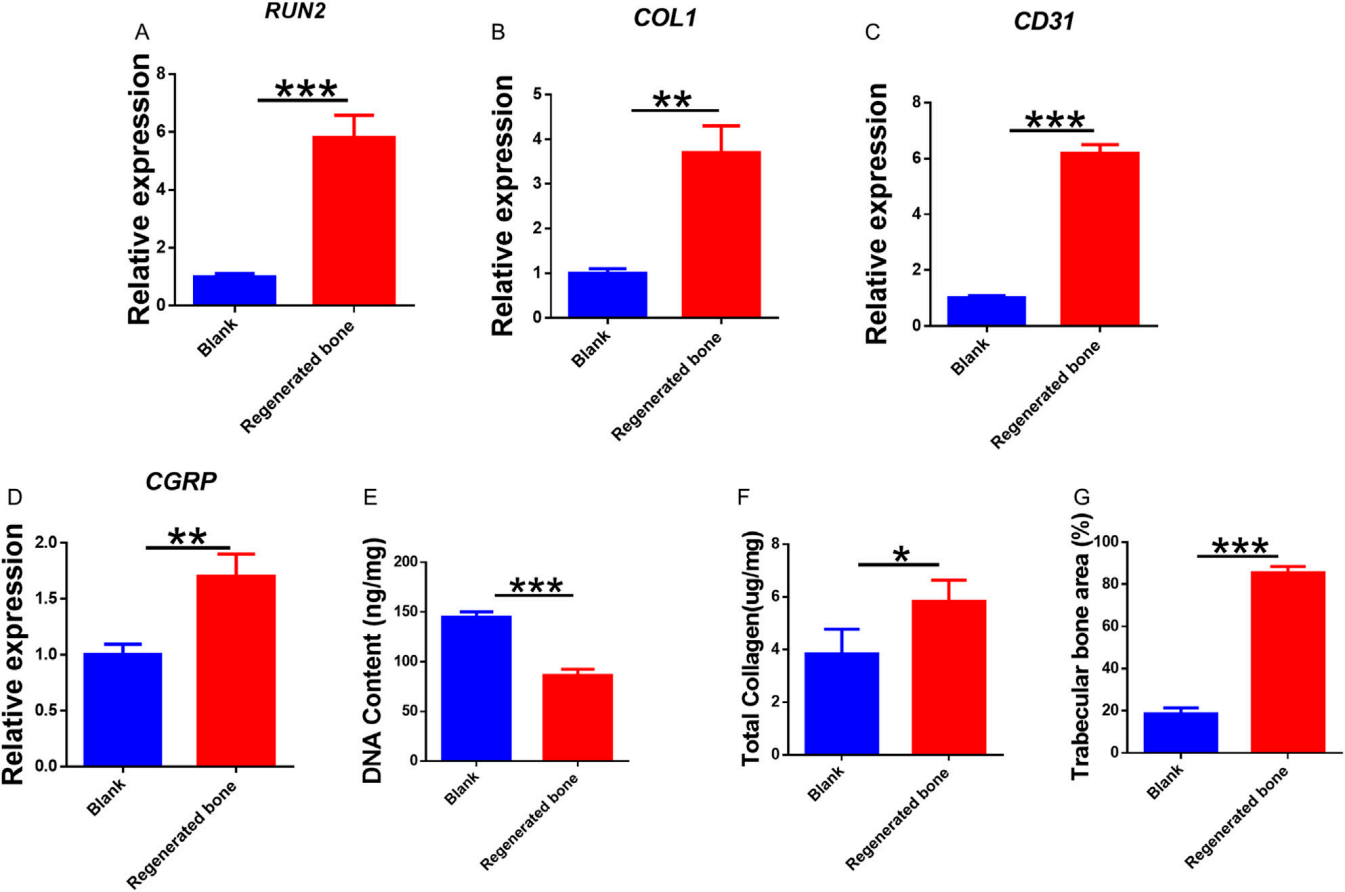
Quantitative analysis of regenerated bone. **(A–D)** Genes expression of *RUNX2* **(A)**, *COL1* **(B)**, *CD31* **(C)**, and *CGRP* **(D)**. Quantitative analysis of DNA content **(E)**, total collagen **(F)**, and trabecular bone area **(G)** in the tow groups.

**FIGURE 10:**
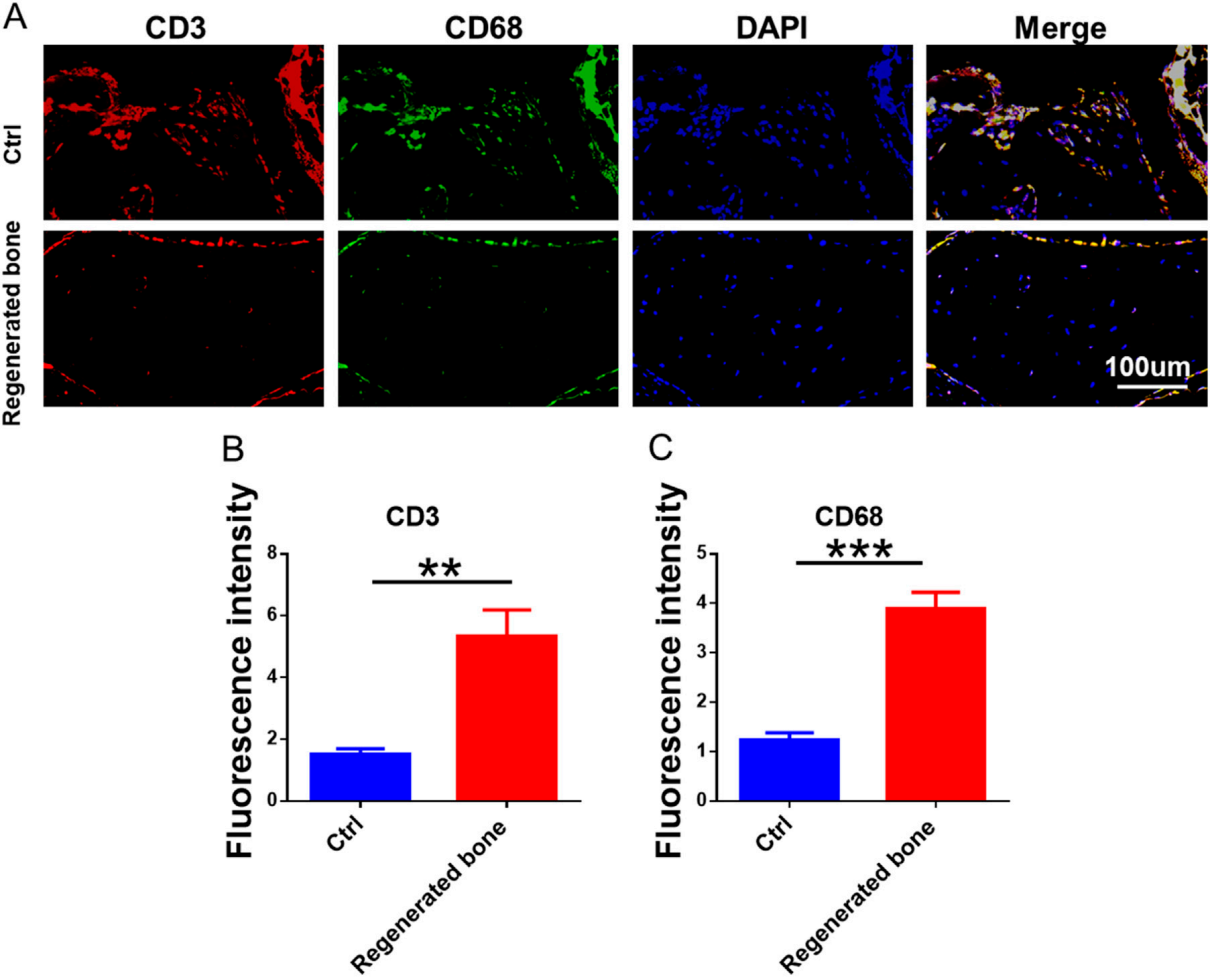
Inflammatory response of regenerated bone. **(A)** The immunofluorescence staining of CD3 and CD68. **(B–C)** The quantitative analysis of fluorescence intensity for CD3 and CD68.

The repair of infected bone defects *in vivo* represented a critical aspect in this study. To investigate the efficacy of BMSCs-loaded imCOL1MA (Engineered bone) in the repair of infected bone defects, we established bone defects in the tibial head of rabbits and subsequently added them with MRSA to induce infection. The control group (Ctrl) received no treatment, while the experimental group was filled with Engineered bone (Figure 7A). Our results indicated that the engineered bone group successfully regenerated new bone characterized by abundant bone trabecular and collagen, whereas the control group predominantly formed fibrous tissue (Figures 7B-C, 9G). Additionally, the expression levels of bone-specific proteins (Figures 8A, C, D) and genes (Figures 9A-C) associated with COL1 and CD31 were significantly higher in the engineered bone group compared to the control group, suggesting that this strategy could effectively promote the bone regeneration. Furthermore, we investigated nerve regeneration within the newly formed bone tissue, revealing that the engineered bone group exhibited a substantial increase in nerve regeneration (Figures 8B,E, 9D). Quantitative analyses of DNA and total collagen further corroborated the superior bone regeneration observed in the engineered bone group (Figures 9E,F). Collectively, these findings indicate that this strategy could achieve rapid neuralized and vascularized bone regeneration. Finally, to assess the inflammatory response, immunohistochemical stainings of CD3 and CD68 were conducted (Figures 10A-C), and the results revealed the engineered bone group significantly reduced the infiltration of macrophages and neutrophils. In summary, we have developed a satisfied strategy for rapid osteogenesis with neurogenesis and angiogenesis in infected bone defect using biomimetic biomineralized and antibacterial engineered bone.

## 3 Conclusion

Infected bone defects represent one of the most prevalent clinical conditions, affecting millions of patients annually. The local infection and necrosis associated with these defects exacerbate the injury, prolong healing times, and result in significant localized pain, presenting a substantial challenge for clinical repair. In this study, we developed a biomimetic mineralized and antibacterial imCOL1MA hydrogel by employing methacrylated COL1, CNBIS, and mMicrospheres, which was further loaded with BMSCs to form osteogenic engineered bone for the repair of infected bone defects. Briefly, we first optimized the concentration of COL1MA for BMSCs survival, then adjusted proportion of CNBIS to create an appropriate osteoinductive microenvironment, and encapsulated Magainin II in poly (lactic-co-glycolic acid) (PLGA) microsphere for long-term antimicrobial function. Consequently, the promising mineralized and antibacterial imCOL1MA was prepared using 10% COL1MA, 2% CNBIS, and 1% mMicrospheres. The imCOL1MA scaffold served as significant antimicrobial efficacy, excellent biodegradability, good biocompatibility, and osteoinductive microenvironment. As a result, the engineered bone could achieve rapid (only 4 weeks) vascularized and neuralized bone regeneration in a rabbit model of infected bone defects. Therefore, this strategy proposed a significant advancement for addressing the multifaceted challenges of infection-related bone defects, offering a clinically translatable method for rapid vascularized and neuralized bone repair. However, the current repair strategy still need further investigation involving larger infected bone defects in large animals to evaluate its preclinical safety.

## 4 Materials and methods

### 4.1 Preparation of COL1MA

Initially, 1 g type I collagen (Macklin) was dissolved in 100 mL deionized water. Subsequently, 1 mL methacrylic anhydride (Aladdin) was added dropwise to the solution, and sodium hydroxide solution was added to adjust the pH to 8-9. After a 12-h reaction under ice conditions, dialysis was performed, and the solution was subsequently lyophilized form dried COL1MA. The COL1MA precursor was prepared by mixing 3% lithium phenyl-2,4,6-trimethylbenzoylphosphinate (LAP, EFL) with the lyophilized COL1MA at 65°C, which was further cross-linked by illumination with 365 nm UV light to form the COL1MA hydrogel.

### 4.2 Preparation of iCOL1MA

Inspired by the composition and proportion of natural bone inorganic salts, we first mixed 84% hydroxyapatite (Duoxi, H24M10Z89099, 5-20um), 10% calcium carbonate (Sinopharm Chemical Reagent, 20240730), 2% magnesium phosphate (MACKLIN, C11161191), 2% calcium citrate (Sangon, D105BA0056), and 2% disodium hydrogen phosphate (Sinopharm Chemical Reagent, 20241014) in a proportional way to form composite native bone inorganic salts (CNBIS). And then the CNBIS was added to the COL1MA precursor to prepare iCOL1MA hydrogel under the same conditions used for forming COL1MA hydrogel.

### 4.3 Preparation of imCOL1MA

The preparation of Magainin II-loaded microspheres involved several key steps. Initially, 10 mg Magainin II was dissolved in 300 µL deionized water. Concurrently, 400 mg of PLGA (30,000 kD, hydroxyl: carboxyl = 3:1, hydroxy-terminated, Sangon) was dissolved in 4 mL of dichloromethane (Sangon). The Magainin II solution was then added into dichloromethane solution to create a homogeneous suspension. This suspension was subsequently added dropwise to a solution of 2% polyvinyl alcohol (80-120 kD, Solarbio), followed by the evaporation of dichloromethane. The microspheres were then centrifugated and lyophilized for future use. Finally, the lyophilized microspheres were incorporated into iCOL1MA to form imCOL1MA. To evaluate the sustained release characteristics of microspheres, BSA microspheres were prepared using the same methodology. The 50 mg BSA microspheres were put in 5 mL of phosphate-buffered saline (PBS) and incubated at 37°C with shaking at 120 RPM for durations of 1, 3, 5, 7, 14, 21, 28, 35, 42, 49, and 56 days, respectively. The release of the microspheres was quantified using an Elisa kit (Merck).

### 4.4 Mechanical strength test

The samples were prepared as original pieces with a diameter of 8 mm and a thickness of 3 mm. They were tested using a mechanical testing machine (GT-TCS-2000 single-column apparatus with a 100 N capacity) at a compression rate of 1 mm/min. The stress-strain curve was obtained from these tests, and the compression modulus was calculated from the curve, with n = 3.

### 4.5 Antibacterial test

Methicillin-resistant *Staphylococcus aureus* bacterial broth was diluted to 10^6 CFU/mL using a sterile PBS solution. Subsequently, 100 μL of this dilution was uniformly coated onto luria-bertani (LB) solid medium. The sterilized samples were separately put onto the surface of the medium without any additional treatment. The dishes were then placed in a constant temperature incubator set at 37°C for 24 h. Following the incubation period, the bacteria were removed, and images were captured using a standard camera to measure the size of the inhibition zones.

### 4.6 Structure and elemental analysis of imCOL1MA

The lyophilized COL1MA, iCOL1MA, mMicrosphere, and imCOL1MA were initially plated onto a conductive gel platform for gold spraying. The surface structure was then examined using scanning electron microscopy (SEM, Hitachi Regulus 8100). Finally, elemental analysis was conducted based on backscattered electrons.

### 4.7 Isolation and culture of bone marrow mesenchymal stem cells (BMSCs)

New Zealand white rabbits (8 weeks, 1–1.5 kg) were anesthetized and their skin was prepared and sterilized for havesting the bone marrow. Subsequently, 10 mL bone marrow was extracted and 40 mL of DMEM medium containing 10% fetal bovine serum and 1% antibiotics was added to the bone marrow, which was then mixed and transferred to a culture dish for 5 days. The medium was then changed every 3 days until the cells reached full confluence. During the subculture process, the medium was first discarded, and any excess was washed off using PBS. Trypsin was then introduced to facilitate digestion. After complete digestion, additional medium was added for final processing, and the resulting cell suspension was collected and centrifuged for re-plating and further culture.

### 4.8 CCK-8

100 µL of BMSCs suspension (1 × 10^7^/mL) was added to each well. After a 24-h incubation period, the absorbance of the supernatant medium was measured according to the instructions provided with the CCK-8 kit (DOJINDO) to assess cell proliferation.

### 4.9 Live/dead staining

BMSCs were introduced into the imCOL1MA hydrogel at a concentration of 3 × 10^7^/mL to form engineered bone. Subsequently, the engineered bone were cultured in a 37°C incubator for 7 days. The biocompatibility of the scaffolds was assessed using the Live/Dead assay kit (DOJINDO).

### 4.10 RT-qPCR

Samples were placed in TriZol (Invitrogen) within 30 min of collection, and total RNA was extracted using a total RNA extraction kit. cDNA was synthesized using the RNA reverse transcription kit (Evo M-MLV RT Kit for qPCR AG11707, Accurate Biology, China), after which amplification reactions were performed using an Applied Biosystems AB instrument (Foster City, CA). β-Actin served as an internal control, and all relevant primers were designed and synthesized by Bioengineering (Shanghai) Co., LTD.

### 4.11 Animals and ethics

New Zealand white rabbits (8 weeks, 1–1.5 kg) were purchased from the Shanghai Jiangnan Experimental Animal Technology Co., Ltd. (Shanghai, China). All animal procedures were performed in accordance with the Guidelines for Care and Use of Laboratory Animals of Shanghai Jiao Tong University and approved by the Animal Ethics Committee of Shanghai Jiao Tong University (A2023069).

### 4.12 Surgery and infectious defect models

A bone defect with 5 mm in diameter and 4 mm in depth was created in the lateral condyle of the rabbit tibial capitulum. Subsequently, 100 µL methicillin-resistant *S. aureus* suspension (10^6^ CFU/mL) was added to the defect to prepare an infectious defect. The control group received no treatment, while the experimental group was treated with functional engineered bone. Four weeks later, the rabbits were euthanized for regenerated bone harvesting.

### 4.13 Histological staining

After 4 weeks of *in vivo* repair, rabbits were anesthetized, and regenerated bone samples were collected. The samples were fixed with 4% paraformaldehyde for 24 h, followed by decalcification using an EDTA rapid decalcification solution (Shanghai Daixuan Biotechnology Co., LTD.). Subsequently, the samples were dehydrated, embedded, and sectioned for further analysis. Conventional hematoxylin and eosin (H&E) staining and Masson staining were employed to observe the structure and collagen content of the regenerated bone. Additionally, immunofluorescence staining for COL1 (GB11022), CD31 (ab28364), CGRP (ab283568), CD3 (ab16669), and CD68 (ab283654) was performed to assess collagen, vascularization, nerve presence, and inflammatory infiltration in the regenerated bone.

### 4.14 Quantitative analysis

After the rabbits were euthanized, fresh samples were obtained, and the regenerated bone tissue was excised and placed in PBS for subsequent analysis. The digestion of the samples was conducted using proteinase K (20 μg/mL, Solarbio) at 56°C, followed by quantification of DNA content using the Quant-iT PicoGreen dsDNA Kit (Invitrogen). Total collagen levels were assessed using the hydroxyproline (HYP) assay kit (Sigma-Aldrich).

### 4.15 Statistical analysis

Statistical analyses were conducted using GraphPad Prism 6, and the results are presented as mean ± standard deviation. Comparisons between two groups were performed using the t-test, while multiple comparisons were analyzed using one-way ANOVA. * represents P < 0.05,** represents P < 0.01, and *** represents P < 0.001.

## Data Availability

The original contributions presented in the study are included in the article/supplementary material, further inquiries can be directed to the corresponding author.
